# A conceptual review on action-perception coupling in the musicians’ brain: what is it good for?

**DOI:** 10.3389/fnhum.2014.00603

**Published:** 2014-08-21

**Authors:** Giacomo Novembre, Peter E. Keller

**Affiliations:** Marcs Institute - University of Western SydneySydney, NSW, Australia

**Keywords:** action-perception coupling, music, training, prediction, joint action

## Abstract

Experience with a sensorimotor task, such as practicing a piano piece, leads to strong coupling of sensory (visual or auditory) and motor cortices. Here we review behavioral and neurophysiological (M/EEG, TMS and fMRI) research exploring this topic using the brain of musicians as a model system. Our review focuses on a recent body of evidence suggesting that this form of coupling might have (at least) two cognitive functions. First, it leads to the generation of equivalent predictions (concerning both when and what event is more likely to occur) during both perception and production of music. Second, it underpins the common coding of perception and action that supports the integration of the motor output of multiple musicians’ in the context of joint musical tasks. Essentially, training-based coupling of perception and action might scaffold the human ability to represent complex (structured) actions and to entrain multiple agents—via reciprocal prediction and adaptation—in the pursuit of shared goals.

## Introduction

In recent years, psychological (Prinz, 1997, 2013), neurophysiological (Rizzolatti and Craighero, 2004; Rizzolatti and Sinigaglia, 2010) and computational (Wolpert and Kawato, 1998; Wolpert and Ghahramani, 2000) accounts have suggested that action perception and action execution are intrinsically coupled in the human brain. Given an association between movements and their ensuing effects, the perception of an effect can trigger a representation of the movement necessary to execute it. And vice versa, movement can trigger perceptual processes.

The roots of the concept of action-perception coupling can be traced to 19th century theorizing on the ideo-motor principle, which holds that actions are triggered automatically by the anticipation of their intended perceptual effects (e.g., Lotze, 1852; James, 1890; for reviews, see Koch et al., 2004; Stock and Stock, 2004; Shin et al., 2010). These roots lay buried beneath the blanket of behaviorism until they received renewed nourishment by Sperry (1952) work on perception-action cycles in the nervous system and then a wave of interest in cognitive mechanisms of intentional action control from the late 1960s to the 1980s (e.g., Greenwald, 1970; Prinz, 1987). This wave culminated in Prinz’s (1990) proposal that perception and action are coded in a common representational domain, and are therefore linked by shared neural resources. The subsequent discovery of “mirror neurons” in the macaque monkey (Gallese et al., 1996; Rizzolatti et al., 1996) provided a potential neurophysiological basis for these proposed links and ushered in an era of intense investigation of the so-called “mirror system” in the human brain (Rizzolatti and Sinigaglia, 2010).

While the notion of a unifying mechanism for action perception and execution has profound implications for human cognition, the goals and functioning of such a mechanism have not yet been fully understood. A potential avenue of research that has the potential to shed light upon this issue is the study of individuals who mastered a certain sensorimotor task, such as expert musicians, whose brain is an excellent example of action-perception coupling where movements and intended sounds become strongly associated after long-term musical training (Zatorre et al., 2007; Herholz and Zatorre, 2012).

Let us take a basic example: striking a piano key with a finger. The movement (striking the key) is intended to generate a goal (a piano tone). When this is observed from the “outside” perspective of another individual, this phenomenon seems straightforward: the movement preceded its goal. However, when considering a “first person” perspective, it is the musician’s intention (i.e., producing a piano tone) that leads the generation of a movement: moving the finger toward the piano key. This distinction might seem trivial, but in fact it represents a fundamental step to understanding that movements and their ensuing effects are intrinsically *coupled* in the human brain and in cognition. More specifically, a representation of a perceptual effect can trigger the movement necessary to produce the effect itself.

The present article aims at providing a “conceptual” review of action-perception coupling in the musicians’ brain. First, we will review evidence showing how the coupling between action and perception strengthens as a result of musical training. Secondly, we will focus on a selected body of studies that—in our opinion—shed light upon the *functional and cognitive relevance* of action-perception coupling, in other words: what it is good for.

**Figure 1:**
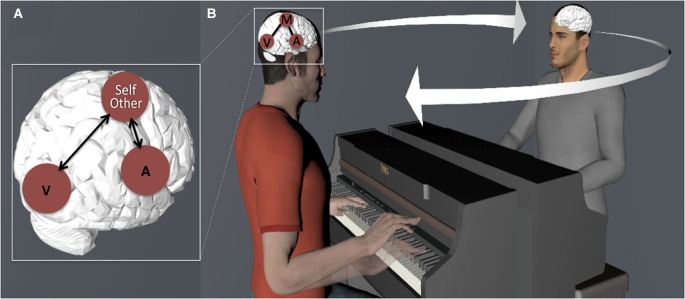
**(A)** Schematic illustration of coupling between sensory (A—Auditory and V—Visual) and motor cortices in the musician’s brain. **(B)** Action-perception coupling is used as a resource for generating predictions (note the future state of the hand of the pianist seated on the left) and integrating representations of self and other-related actions, leading to entrainment of multiple individuals’ brains and behavior.

We will emphasize how this coupling can be used for (1) generating predictions of our own as well as others’ (i.e., observed) actions; and (2) as a resource for the co-representation of and coordination with other musicians in the context of an inter-action (or joint action) (see Figure 1). These combined functions highlight the fundamental role that action-perception coupling plays in interpersonal entrainment, that is, the spatiotemporal anticipation and coordination between two or more individuals engaged in rhythmic behavior (Keller et al., in press; Phillips-Silver and Keller, 2012).

The focus of our review is on neuroimaging studies (fMRI, M/EEG, TMS) that place special emphasis on (sensory-) motor and cognitive mechanisms, mostly conducted with musically-trained participants (or directly relevant for them), with also some references to purely behavioral investigations.

## Action-perception coupling in the musicians’ brain

The highly plastic nature of the musician’s brain has been emphasized in the literature in recent years (see Pascual-leone, 2001; Zatorre et al., 2007; Herholz and Zatorre, 2012). Here, we will focus on those studies that specifically addressed action-perception coupling in musicians or in individuals who received musical training for experimental purposes. We will first mention select behavioral investigations (Section Behavioral Evidence), and then move on to neuroimaging evidence from studies using hemodynamic measures (fMRI) and electrophysiological techniques (EEG, MEG), as well as brain stimulation methods (TMS; Section Neuroimaging Evidence).

### Behavioral evidence

Research in experimental psychology has explored action-perception links in music through the use of action-effect compatibility manipulations. Drost et al. (2005a,b) compared pianists and non-musicians in the context of an interference paradigm where participants had to play a chord on a piano in response to visual imperative stimuli. These visual stimuli were accompanied by simultaneously presented task-irrelevant sounds, which could either match or not match the target chord. It was found that incongruent sounds delayed execution time in pianists but not non-pianists (Drost et al., 2005a). In addition, these incongruent sounds tended to induce false responses, i.e., production of the heard chord, instead of the imperative one (Drost et al., 2005b). In a subsequent study requiring pianists and guitarists to play chords on their instrument, the interference effect was only observed when the timbre of the musical sound matched the participant’s instrument (Drost et al., 2007). The studies by Drost and colleagues demonstrate that auditory perception primes action if strong action-perception links have been established through instrument-specific training.

Analogous conclusions can be drawn from studies testing the interference between passive music perception and general (i.e., not musical) actions. For instance, Taylor and Witt (2014) had pianists and novices listening to task-irrelevant ascending or descending scales while making left or right arm movements (to press one of two buttons in response to a visual target). Because the piano canonically has keys increasing in pitch from left to right, rightward movement along the keyboard is associated with ascending pitch and leftward movement with descending pitch (Lidji et al., 2007). Accordingly, Taylor and Witt (2014) found that pianists but not the novices responded faster when the direction of their response was compatible with the direction of the musical scale heard in the background (i.e., right-button responses were faster during ascending scales and vice versa).

In related research, Keller and Koch (2006a) found that mental images of anticipated action effects can prime responses to a similar degree as is observed with congruent and incongruent sounds, highlighting the role of action-perception coupling in action preplanning (i.e., before sounds are actually perceived). Subsequent studies investigated such preplanning in sequential actions—a definitive aspect of music performance—by requiring participants to respond to visual imperative stimuli by producing series of finger taps on vertically aligned keys. Taps triggered tones in some conditions, where the key-to-tone mapping was manipulated (between blocks of trials) to be either congruent or incongruent in terms of pitch and spatial height. One version of this task (Keller and Koch, 2008) required participants to respond as quickly as possible to the imperative stimuli. Results indicated that reaction times were shorter in conditions where sequences of finger taps and tones were congruent in “height” than when they were incongruent. This effect was restricted to musicians and, furthermore, increased in size with years of musical experience. Therefore, action-perception coupling associated with musical training allowed participants to plan their actions by imagining the auditory sequences in an anticipatory fashion, and the efficiency of such preplanning was greatest when movements and their auditory effects were congruent. Further studies using a version of the paradigm that required taps to be produced at a specific tempo (rather than rapidly) demonstrated that action-perception coupling does not only enhance the efficiency of action planning, but also facilitates timing accuracy and economical force control by optimizing movement kinematics (Keller and Koch, 2006b; Keller et al., 2010).

The importance of action-perception coupling in action planning has also been explored by a series of studies where auditory feedback was varied during piano performance (Pfordresher, 2005; Pfordresher and Palmer, 2006). Participants with variable levels of musical training performed simple melodies from memory on the piano while the auditory feedback (i.e., the musical pitch) was altered. One form of alteration involved serially shifting the pitch sequences relative to keystroke sequences that would normally produce them (e.g., each keystroke triggered the tone typically associated with the previous keystroke, in which case the produced tone sequence was similar to the learned sequence, but at a lag) or by using random key-to-tone assignments. Pfordresher (2005) hypothesized that the serial shift would lead to higher error rate production (compared to the random shift or a non-altered condition) to the extent that action planning is underpinned by shared representations of movements and their effects. Consistent with this hypothesis, it was observed that the majority of the participants performed the highest number of errors when the auditory feedback was serially shifted. Further experiments indicated that the same effects could also be observed with non-pianists (who were trained to produce simple melodies) and with individuals having small amounts of musical training (4.7 years), who learned to produce the melodies without auditory feedback. This indicates that the ability to form cross-modal congruency is not a music-specific ability, but rather a domain-general one, highlighting the human predisposition for developing strong sensory-motor associations through specialized experience such as musical training.

Taken together, this behavioral evidence indicates that the perception or mental imagery of sounds—which would normally be associated with specific movements—trigger representations of those specific movements.

### Neuroimaging evidence

Neuroimaging research has shed light on the neurophysiological mechanisms underpinning action-perception coupling in the musician’s brain. Haueisen and Knösche (2001) conducted a magnetoencephalography (MEG) study that allowed them to investigate brain responses to familiar piano pieces in musicians with or without piano experience. In piano players, perception of these pieces led to an increase of neural activity over the motor cortex hand area. Most interestingly, the authors found a distinct spatial response to notes that would be preferably played with the thumb vs. the little finger, which matched the homuncular organization of the primary motor cortex (M1). The finding that the acoustic perception of music within an individual’s behavioral repertoire lead to an increase of motor cortical activity in musicians has been replicated in other neuroimaging studies using different methods. For example, D’Ausilio et al. (2006) used Transcranial Magnetic Stimulation (TMS) to trigger Motor Evoked Potentials (MEP) in a forearm muscle normally used to play the piano. Cortico-spinal excitability (which was indexed by the amplitude of the MEPs) was found to increase while pianists listened to a rehearsed piano piece compared to an unrehearsed one. Moreover, Bangert et al. (2006) ran an fMRI study where professional pianists and nonmusicians heard novel piano sequences that were synthesized online (and therefore could not be familiar). Compared to non-musicians, professional pianists showed a broad network of motor areas responding to the piano sequences, including both primary motor and premotor (BA 4/6) regions. To explore whether this auditory-to-motor transformation was bidirectional, the authors also examined the effect of producing piano tones in the absence of auditory feedback. This latter task led to the activation of auditory-related brain regions, including the superior temporal gyrus (BA 22).

Motor activations in the musician’s brain are not only elicited by the acoustic presentation of music, but also visual presentations of musical actions. In two fMRI experiments, Haslinger et al. (2005) and Hasegawa et al. (2004) presented video recordings of hands playing a silent keyboard. Despite the fact that these videos were mute (i.e., no sounds were presented), the authors observed the activation of a fronto-parietal brain network—including premotor cortex (BA6) and inferior parietal cortex—which was very similar to the one revealed in the study by Bangert et al. (2006) (who presented sounds rather than silent videos).

Besides auditory- and visual-motor coupling, further forms of coupling link motor processes with tactile, proprioceptive and haptic sensory feedback (i.e., striking a piano key with your finger would normally be associated with the experience of proprioceptive feedback from the moving fingers, as well as, possibly, the sensation of the movement of the piano relative to the pianist). This research avenue has not yet been explored extensively in the musicians’ brain. However, evidence of enhanced audio-tactile integration in trained musicians (Schulz et al., 2003; Kuchenbuch et al., 2014), as well as general claims of somatosensory and motor processes being inter-dependent (see e.g., Keysers et al., 2010 or van Ede and Maris, 2013), suggest that findings analogous to those outlined above might be reported in the future.

Taken together, these data indicate that musical training leads to the emergence of cross-modal action-perception coupling, where perception of the effects of musical actions (either the sounds produced or the visual presentation of the movement patterns) triggers a representation of the movements necessary to produce these effects. This idea has profound implications for human cognition more broadly, because it could be applied to other motor tasks and therefore generalize across individuals with different types of experience. For this reason, other research has investigated to what extent these results could be replicated in naïve participants who received musical training only shortly before taking part in an experiment. Lahav et al. (2007) trained non-musicians to play a piano piece by ear (without notation) over a period of 5 days. Following the training, participants were presented with either the trained pieces, untrained pieces (having the same notes, but in a different order) or familiar but motorically unknown pieces. Remarkably, activation of the frontoparietal motor-related network discussed above (here comprising Broca’s area, the premotor region, the intraparietal sulcus, and the inferior parietal region) increased most strongly for the trained pieces, weakly for the untrained ones, and not at all for the motorically unknown pieces (relative to a rest baseline condition). Thus, a few days of training were sufficient to replicate the effects—previously described in studies with experienced musicians—in a group of non-musicians (see also Bangert and Altenmüller, 2003, for earlier EEG evidence consistent with this).

A handful of recent studies have made noteworthy progress towards understanding the functioning of this action-perception coupling mechanism, and how it emerges through learning. Engel et al. (2012) trained non-musicians to play melodies either by ear (and without seeing their hands) or by imitating visual movement patterns (without auditory feedback). Following training, participants were able to recognize melodies learned in one modality upon presentation in the other (i.e., untrained) modality. However, recognition accuracy and fMRI data indicated the cross-modal transfer was stronger when the melodies had been trained by ear. Moreover, in order to demonstrate that sensory-motor coupling emerges as a result of motor learning, and not visual familiarity, Candidi et al. (2012) trained non-musicians to recognize piano fingering errors during the visual presentation of silent musical sequences. Expert pianists showed a somatotopic corticospinal facilitation—indexed by the amplitude of MEPs triggered by TMS—of the finger that committed the error (consistently with the study by Haueisen and Knösche, 2001, who also reported finger-specific activations, but in response to acoustically presented music). Visually trained non-musicians, however, did not show the same facilitation effect, although they were equally able to recognize the errors. Thus, visual experience (or auditory experience, cf. Lahav et al., 2007) is not sufficient to recognize movement patterns if motor learning has not taken place.

Taking together the studies reviewed above, there is converging evidence from both behavioral and neurophysiological methods (including EEG, MEG, TMS and fMRI) that, given an association between movements and their ensuing effects, the perception of an effect can trigger a representation of the movement necessary to execute it. The musician’s brain is an excellent example of action-perception coupling because movements and intended sounds become strongly associated after long-term musical training. On the behavioral level, it has been consistently shown that the representation of a musical sound and the motor resources necessary to perform the sound are represented by a comparable code and can interfere between each other (Drost et al., 2005a,b, 2007; Pfordresher, 2005; Keller and Koch, 2008; Taylor and Witt, 2014; see also Koch et al., 2004). On the neural level, listening to a trained musical sequence activates the motor brain areas necessary for executing it, as evidenced by measures such as corticospinal excitability (D’Ausilio et al., 2006), blood-oxygen-level-dependent (BOLD) signal (Bangert et al., 2006; Lahav et al., 2007), EEG potentials (Bangert and Altenmüller, 2003) and MEG fields (Haueisen and Knösche, 2001). Conversely, the visual perception of (silent) musical actions leads to similar brain co-activations (Hasegawa et al., 2004; Haslinger et al., 2005; Engel et al., 2012; Candidi et al., 2012), demonstrating that action-perception coupling in the musicians’ brain is multimodal (i.e., visual and auditory) (see Figure 1A). Additional research has shown that these coupling effects can also result after a short period of musical training (with naïve participants), implying that such action-perception matching system is not necessarily music-specific, but rather stands as task-specific example of a cognitive mechanism with broader relevance (Bangert and Altenmüller, 2003; Lahav et al., 2007; Chen et al., 2012; Engel et al., 2012). Moreover, having only visual (Candidi et al., 2012) or auditory (Lahav et al., 2007) experience with a given action is not sufficient to trigger these motor responses—active motor learning is necessary.

## The predictive character of action-perception coupling

### Prediction of self-generated actions (and effects)

The studies reviewed above were not designed to address the temporal dynamics of action-perception coupling in the brain, but this aspect is fundamental in understanding its cognitive and behavioral relevance.

Let us return to the example of the finger striking a piano key (movement) to generate a sound (goal) (see Introduction). As we noted, from a first-person perspective, it is the musician’s intention (i.e., to produce a piano tone) that leads to the execution of a movement. Given this, one would hypothesize that—in the musician’s brain—the two processes associated with intending to perform a specific keystroke, and hearing the auditory feedback, are at least in part independent and have different priorities, i.e., the actual sound of the key should be predicted on the basis of its preceding “intended” neural representation.

Following this reasoning, Maidhof et al. (2009) and Ruiz et al. (2009) conducted two similar EEG studies in which they examined the ERPs preceding the execution of piano errors. Both these studies reported behavioral and electrophysiological markers of performance errors. First, erroneous keystrokes were produced with less force, and therefore generated a softer sound (which might be taken as an index of uncertainty, see Keller, 2012b). Second, an early negative deflection (or Error Related Negativity, ERN) of the EEG signal was found to anticipate the actual mistake by 100 ms (Maidhof et al., 2009) and 50–70 ms (Ruiz et al., 2009). Source localization analysis revealed that these responses were generated by the anterior cingulate cortex (a brain region implicated in action monitoring, Kiehl et al., 2000; Kerns et al., 2004) and, most interestingly, this effect was independent of whether or not auditory feedback was available (Ruiz et al., 2009). Thus, errors were detected *prior* to their execution, and this occurred independently of whether the pianists could hear the actual feedback of the performance.

This finding is particularly important because it provides evidence that, during performance, internal forward models predict the outcome of ongoing motor commands by comparing them with efference copies (i.e a prediction of the perceptual effects of the motor command) (Wolpert et al., 1995). In other words, during the execution of a musical sequence, images of the “intended” sounds are formed well ahead their generation, and compared in real time with the state of the body. Thus, the coupling of sensory and motor cortices is a dynamical process with a strong anticipatory character that, given the existence of an association between movements and their ensuing effects, permits the generation of predictions about the state of our own body and the sensory consequences of our movements.

Further evidence has supported the notion that internal models play a role in real-time prediction during online action planning. Maidhof et al. (2010) compared EEG brain responses to expectancy violations in musical action (i.e., during piano performance) and perception (i.e., during listening). Both types of violation led to a negativity peaking at around 200 ms after tone presentation. However, the amplitude was larger for the action violation (i.e., when auditory feedback was lowered by the interval of one semitone for a single keystroke) compared to the perceptual violation (i.e., while listening to the same lowered interval), indicating that the expectations associated with the intention to produce a tone override those based on perceptual processes alone. This notion is corroborated by evidence showing that motor training of a specific melody enhances auditory expectancies by amplifying neural electrophysiological potentials arising from cortical motor structures (both premotor and supplementary motor regions) (Mathias et al., 2014). Furthermore, Ruiz et al. (2011) explored EEG oscillatory markers predicting an error during musical performance. It was shown that a burst of beta band oscillations (an electrophysiological marker of motor processes, Salenius and Hari, 2003; Feurra et al., 2011) that originated from the posterior fronto-medial cortex (pFMC, which includes the anterior cingulate cortex, cf. Ruiz et al., 2009) anticipated the error by 120 ms. Moreover, the efficiency of motor control correction mechanisms, i.e., the reduction of the force utilized to execute a wrong note (cf. Maidhof et al., 2009; see Keller, 2012b), could be predicted based on the beta band synchronization between pFMC and brain regions implementing control adjustments (i.e., lateral prefrontal cortex) (Ruiz et al., 2009). These electrophysiological findings are further corroborated by fMRI evidence demonstrating that the alteration of pitch feedback during piano performance modulates the BOLD signal within the anterior cingulate cortex, as well as in motor regions such as the cerebellum and the supplementary motor area (Pfordresher et al., 2014). Taken together, these data indicate further that musical training leads to a tight coupling between sensory and motor cortices, and that this coupling might underpin the generation of sensory predictions—based on internal models—*within* the musician’s brain.

### Prediction of actions produced by others

A remarkable property of this dynamical process is that it does not only permit predictions of our own movements, but can also be used to generate predictions about others’ actions (see Wolpert et al., 2003; Kilner et al., 2007a,b; Overy and Molnar-Szakacs, 2009, for relevant computational, neurophysiological and cognitive models). Lee and Noppeney (2011) designed an elegant study that combined psychophysics and fMRI to investigate the temporal binding between sensory and motor processes in musicians and non-musicians. Participants were required to attend to musical and speech stimuli in which the synchrony between sounds and images (of either a speaking face of a hand playing the piano) was manipulated parametrically. As could be expected, the two groups were equally sensitive to the temporal asynchronies in the speech domain, but the musician group was superior in detecting temporal asynchronies in the musical domain. Dynamic causal modeling revealed that this superior performance was associated with greater effective connectivity within a network of brain regions including the superior temporal sulcus, the premotor cortex and the cerebellum. Thus, cross-modal plasticity due to musical training (as reviewed in Section on Action-Perception Coupling, Neuroimaging evidence) led to the fine-tuning of internal forward models (see previous section above) that, critically, permit the generation of predictions of observed actions with high temporal resolution. Accordingly, coupling emerges within an individual brain, but can also be used to generate predictions about others’ actions.

These fine-tuned internal forward models might allow a musician to predict not only *when* an event will occur, but in some cases also *what* event will occur. Through training, the musicians’ brain does not only bind specific events across sensory and motor modalities. In addition, the brain learns which successions of tones are most likely to occur according to regularities associated with the rules that govern harmony (i.e., sequential chord progressions) in a given musical tradition. This phenomenon has been studied for some time in the context of purely auditory perceptual experiments. Participants with and without musical background were presented with sequences of chords that did or did not contain a violation of harmonic structure, while Evoked Response Potentials were measured using EEG. In a series of experiments (for review, see Koelsch and Siebel, 2005; Koelsch, 2012), it was shown that the perception of a harmonic violation led to an early right anterior negativity (ERAN) peaking at around 200 ms after chord presentation. By comparing expert musicians with novices, it was further shown that the amplitude of this negativity was larger for expert musicians (Koelsch et al., 2002). This finding indicates that musical experience leads to the generation of stronger expectancies in the perceptual domain. It should be noted, however, that expert musicians acquire these rules not only by means of perceptual exposure (as a naïve listener cf. Koelsch et al., 2000), but also by means of intensive practice. Therefore, given the tight functional link between sensory and motor cortices highlighted by previous studies (see above), it remained to be explored to what extent expectancies in the auditory domain extend to the motor domain.

Two studies examined this issue. First, in a behavioral study, Novembre and Keller (2011) presented expert pianists with silent videos displaying a musician’s hand performing mute sequences, including occasional chords that were harmonically incongruent with the preceding musical context. The pianists were asked to imitate the chords as quickly and accurately as possible. It was shown that, despite the absence of auditory feedback, imitation was faster and more accurate for chords that were preceded by a congruent context. This result suggests that the harmonic rules implied by the observed actions induced strong expectancies that influenced action execution (cf. Hasegawa et al., 2004; Haslinger et al., 2005). Thus, this study provided first behavioral evidence in favor of harmonic structures regulating not only perceptual processes (as shown by the previous studies, Koelsch et al., 2000, 2002), but also the motor processes involved in producing these structures. This finding was replicated in a subsequent study (Sammler et al., 2013) where EEG was recorded during task performance. ERP data revealed a negativity following the presentation of the final sequential chord, and anticipating chord imitation. The negativity resembled both the ERAN that follows auditory presentation of a harmonically incongruent chord (Maess et al., 2001; Koelsch et al., 2002) and the ERN that anticipates keystroke errors in piano performance (Maidhof et al., 2009; Ruiz et al., 2009, see previous section above). These findings are particularly noteworthy in that they offered the first evidence that the well-known predictive character of the motor system is strongly based on musician’s knowledge of harmonic principles. This indicates that the motor system predicts not only when an action will occur, but also what kind of action will occur. Rule-based predictions in the motor system are consistent with other accounts postulating a sensorimotor processing of syntax, including harmony (Fadiga et al., 2009; Pulvermüller and Fadiga, 2010; see also Molnar-Szakacs and Overy, 2006).

In conclusion, the studies reviewed in this section indicate that the coupling between sensory and motor cortices underpins predictive computations by means of internal models. The studies by Maidhof et al. (2009, 2010) and Ruiz et al. (2009, 2011) explored this notion within the musician’s brain by looking at the relationship between intended sounds and executed movements. The studies by Lee and Noppeney (2011), Novembre and Keller (2011), and Sammler et al. (2013) examined the prediction of other musician’s musical actions. Taken together, the results of this research suggest that musical training leads to the emergence of a sensorimotor system that generates predictions about the identity and timing of upcoming events (for specific evidence supporting the integration of musical pitch and temporal structure within auditory-motor regions, see Brown et al., 2013). Importantly, the functioning of these predictions about* other musicians’* actions suggests that this mechanism could potentially support real-time inter-action between ensemble musicians, where monitoring and prediction of others’ actions is necessary for the establishing and maintenance of group-level coordination (see Figure 1). Recent research examining this hypothesis will be discussed in the next section.

## Action-perception coupling in joint musical action (or social inter-action)

Recent research has explored to what extent action-perception coupling functions as a resource for the co-representation and the coordination of ensemble musicians in the context of joint musical performance (see Figure 1B). In this area, two fundamental components of inter-personal coordination have received particular attention: those that underpin inter-individual sensorimotor synchronization (i.e., timing functions of the motor system), and those that are relevant for the representation of actions produced by self and others in joint action (i.e., others’ action monitoring and self-other integration). We review a series of behavioral (Section Behavioral Evidence) and neuroimaging (Section Neuroimaging Evidence) studies that addressed how action representations of self and other emerge and are eventually integrated in the context of joint musical actions that require synchronization between multiple individuals playing separate musical parts.

### Behavioral evidence

Behavioral research on music performance suggests that expert ensemble musicians form representations of self and other-related actions, and that these representations are influenced by properties of the individual’s own motor system. Evidence for this comes from studies demonstrating that pianists synchronize better with recordings of themselves than with those of other musicians (Keller et al., 2007) and with pianists who are well matched in terms of preferred performance tempo than with pianists who are less well matched in preferred tempo (Loehr et al., 2011). More recently, it has been shown that practicing a co-performer’s part can in fact be detrimental to interpersonal coordination because in this case predictions about micro-timing in the other’s part are based upon one’s own playing style, which may differ from the co-performer’s style (Ragert et al., 2013). These findings suggest that musicians form representations of others’ parts that are based upon internal models that allow one individual to simulate another’s actions. Thus, the manner in which a performer would execute a given piece strongly influences the way in which the performer synchronizes with another’s performance of the piece. While this suggests that representations of others’ actions are generated by means of (neural) simulation processes (Wolpert et al., 2003; Keller, 2008, 2012a) within one’s motor system, this was not tested directly in the above-mentioned studies as they did not employ brain measures. The hypothesis of a predictive—neural—simulation mechanism operating in the context of joint action (see next section) might directly inform research examining musical ensemble as a model for human non-verbal communication (D’Ausilio et al., 2012; Glowinski et al., 2013; Badino et al., 2014), inter-personal synchronization (Goebl and Palmer, 2009; Ragert et al., 2013) and mutual temporal adaptation (Wing et al., 2014).

### Neuroimaging evidence

In a single-pulse TMS study, Novembre et al. (2012) investigated the representation of self and other-related actions in the context of a musical joint action paradigm involving virtual piano duo performance. Pianists learned to perform several pieces bimanually prior to the experiment. During the experiment, they were asked to perform the right hand part of the piece, while the left hand part was either not performed, or believed to be played by a co-performer hidden behind a screen (while the pianists actually heard a recording). This paradigm was intended to lead to a co-representation of the left-hand part, reflecting either the self or the co-performer. The authors examined action representation processes related to the left-hand part by stimulating the right motor cortex (using single-pulse TMS), and observing changed in the MEPs recorded from the resting left arm (cf. D’Ausilio et al., 2006). Results indicated that MEP amplitude was larger when the participant believed that he/she was interacting with a (hidden) co-performer. Remarkably, this effect persisted in a subsequent session in which neither visual nor auditory feedback from the co-performer were provided (though the participants were led to believe that the hidden co-performer was nevertheless playing), and was larger in individuals possessing stronger perspective taking skills (a subscale of empathy, see Davis, 1980, 1983).

The study by Novembre et al. (2012) indicates directly that ensemble musicians form *motor* representations of their ensemble members in the context of joint action, which is consistent with the behavioral evidence reported above. Moreover, this study suggests that these representations have an intrinsic social component, as (1) perceptual feedback is not a prerequisite for co-representation; and (2) individuals who are more prone to take the perspective of others form stronger co-representations.

A study by Loehr et al. (2013) used a joint musical performance paradigm similar to that employed by Novembre et al. (2012) to investigate self and other monitoring and integration while EEG was recorded from pairs of pianists simultaneously. The pianists learned to play both the left and right-hand parts of musical pieces, and were then asked to perform one part each (while hearing and seeing each other’s actions). The experimenters manipulated the auditory feedback from either pianist by creating a mismatch between piano keystrokes and produced tones. The mismatch either did or did not affect the harmony between the players’ parts, hence permitting the differentiation of processes related to monitoring the self’s performance and the joint action outcome. Altered outcomes elicited a feedback related negativity irrespectively of whether it occurred in the pianist’s own or the partner’s part, and a P300 with higher amplitude when the alteration was related to the pianist’s own part. Crucially, the P300 had higher amplitude if it affected the joint outcome compared to the individual outcome, indicating that this task lead to the emergence of integrated representations of self and other-related actions.

A further study by Novembre et al. (2013) used another modification of the virtual piano duo paradigm (cf. Novembre et al., 2012) to explore the extent to which motor representations of ensemble members support efficient temporal coordination between musicians. To this end, pianists were required to adapt with the right hand to tempo changes contained in a recording of the left hand part. The left hand part either had or had not been practiced before the experiment in order to manipulate whether or not a motor representation was formed (cf. Section on Action Perception Coupling, Neuroimaging evidence). In order to interfere with the representation of the left hand part (which was practiced, but not performed), repetitive (double-pulse) TMS was used to disrupt the neural processing in the right primary motor cortex, and tempo adaptation accuracy was measured following the brain stimulations. It was shown that interfering with the motor representation of the left hand part affected temporal adaptation only when the part had been practiced (and therefore could be motorically represented). Moreover, this interference was stronger in individuals with high perspective taking skills, which is noteworthy given that Novembre et al. (2012) demonstrated that these individuals also form stronger representations of others’ action. This finding is also consistent with other accounts that postulate the relevance of empathic and perspective taking skills in the context of interactions between musicians (see Engel and Keller, 2011; Babiloni et al., 2012; Rabinowitch et al., 2013; Pecenka et al., 2013; see also Thoma and Bellebaum, 2012; Sevdalis and Raab, 2014; Gallese, 2014 for reviews on how empathy might modulate cognitive and neurophysiological mechanisms implicated in action monitoring). Thus, the results of Novembre et al. (2013) provide evidence that motor representation processes might be a means used by musicians to monitor others’ (Novembre et al., 2012; Loehr et al., 2013) and ultimately establish synchrony with one another.

It should be noted that the studies reviewed above (Novembre et al., 2012, 2013; Loehr et al., 2013) used sensorimotor *training* tasks as a means to build representations of self and other-related actions. This is an important detail in that it suggests the particular relevance of the body of studies reviewed earlier in the Section on Action-Perception Coupling, where it was shown that listening to or watching the performance of a trained musical piece leads initially to the formation, and later to the activation, of motor representations in the musician’s brain. Considering this, it appears clear that this recent research extends previous work by showing that the training-mediated coupling of perception and action is not confined to individual behavior, but extends to become inter-individual, as it is used for monitoring and integrating (e.g., timing or combined pitches) the actions of other ensemble members with self-generate actions (see Figure 1).

## Musical vs. finger-tapping tasks: a note on the role of “structure” in prediction and joint action

At this point, it should be noted that the current article has not discussed an important body of research that has investigated the predicting character of action-perception coupling through the use of tasks requiring simple repetitive movements, such as finger taps (as recently reviewed, e.g., in Repp and Su, 2013; Patel and Iversen, 2014). The reason for this omission is that repetitive movements, as employed in finger-tapping tasks, do not capture a crucial component of human action: its structure. By the “structure” of an action, we refer to the fact that most actions performed by humans consist of distinct elements that are organized hierarchically within a sequence (Lashley, 1951; Schmidt, 1975; Rosenbaum, 2009). Action sequences are composed of series of different elements—movements—that can be combined in a potentially infinite number of ways (similarly to morphemes composing a word or words forming a sentence), and that it is the combination of these elements, and the context in which are produced, that determine their meaning. Skilled human action is therefore characterized by the need for correct movement sequencing in addition to timing (see Palmer and Pfordresher, 2003). In joint action, entrainment is thus a spatiotemporal rather than purely temporal phenomenon (Phillips-Silver and Keller, 2012).

When exploring how humans predict others’ actions, or integrate their actions with those of others, conventional finger-tapping tasks requiring synchronization with a sequence of beats address how the brain anticipates *when* an event will occur—but not necessarily *what* event it will be. Here we claim that the “what” component calls for additional sensorimotor resources, specifically for a stronger use of cortical motor areas (as reviewed in Section Neuroimaging Evidence). These cortico-motor activations might be responsible for representing the structure of an action, and therefore providing the observer with the means necessary for predicting the goal of that action and, possibly, coordinating with it (e.g., imitating or complementing that action). Coming back to repetitive movements, it should be noted that such movements can acquire some higher-order temporal structure if, for instance, some events are periodically accented to produce metrical structures comprising a “beat” and higher levels of pulsation that group beats. Hence, our claim is consistent with evidence that the perception of increasing metrical saliency and structural complexity in rhythm leads to greater activation of cortical motor regions including SMA and mid PMC (Chen et al., 2006, 2008; see also Schubotz, 2007; Grahn and Rowe, 2009; Stupacher et al., 2013). However, tapping to a beat still does not fully addresses the brain’s ability to “represent” complex actions (i.e., formed by a combination of different movements)—either associated with the self (produced actions) or others (observed actions). These forms of representation might be of fundamental importance when, in real life situations, actions produced by two (or more) individuals are *complementary* (Newman-Norlund et al., 2007; Bekkering et al., 2009)—rather than identical—and need to be integrated in a way that concerns both the timing and the appropriateness of the movement produced.

Joint musical performance is an excellent model with which to explore both inter-personal coordination and complementarity, because in this context individuals need to establish and maintain inter-personal synchronization while also producing appropriate movements (for triggering specific musical sounds that complement the sounds produced by one or more co-performers). Beside musical scenarios, this is a feature of social interaction that applies to quintessential joint activities such as team sport, dance or conversation as well as more ordinary activities, such as lifting a table or assembling a piece of furniture with another individual (Sebanz et al., 2006; Sebanz and Knoblich, 2009). Similar forms of interaction rely strongly upon predictive behavior and are likely to be supported by mechanisms such as action monitoring, perspective taking or, more generally, the integration of complementary action streams related to the self and others. These mechanisms are better suited to be examined in the context of sequentially structured actions rather than repetitive movements.

## Conclusion

In this article, we addressed the functional relevance of action-perception coupling in the musicians’ brain, i.e., what it is good for. Firstly, we reviewed behavioral and neuroimaging research demonstrating that perceptual (either auditory or visual) and motor processes become strongly coupled in the musician’s brain as a result of learning a specific sensorimotor task (e.g., playing a melody on a piano). This might be interpreted as the brain capacity to represent a perceived action in terms of the neural resources necessary for producing it.

Next, we provided evidence supporting two pivotal functions of this form of coupling. One is concerned with the capacity to generate predictions of our own as well as others’ (i.e., observed) actions (i.e., when and what event would occur). The other, instead, is related to the ability to form representations of actions produced by others’, and integrate them with self-generated actions in real time (i.e., co-representation and coordination with other musicians’ motor output) (see Figure 1).

It was further argued that these processes cannot be completely disclosed via research using paradigms that require simple repetitive movements (e.g., finger taps), as the “structural” component of actions is a fundamental one for exploring processes such as action monitoring, perspective taking or, more generally, the integration of complementary action streams related to the self and others. These mechanisms are likely to support both prediction and inter-action in several real-life situations. We advocate the use of tasks requiring the production of complex musical sequences in real or virtual interaction settings to study these mechanisms.

In sum, we have presented evidence that strong coupling between processes related to perception and action emerges in the human brain as a consequence of learning a sensorimotor task. The functional role of this action-perception coupling is not confined to individual behavior, as it extends to *inter*-individual contexts and is suitable for use in inferring the goal of other agents or inter-acting with them. Action-perception coupling thus constitutes a means for entraining multiple individuals’ brain and behavior, and a fruitful path for the understanding of how multiple agents inter-act (via reciprocal prediction and adaptation) to achieve shared goals.

## Conflict of interest statement

The authors declare that the research was conducted in the absence of any commercial or financial relationships that could be construed as a potential conflict of interest.
